# Contribution of cell blocks obtained through endobronchial ultrasound-guided transbronchial needle aspiration to the diagnosis of lung cancer

**DOI:** 10.1186/1471-2407-12-34

**Published:** 2012-01-21

**Authors:** José Sanz-Santos, Pere Serra, Felipe Andreo, Maria Llatjós, Eva Castellà, Eduard Monsó

**Affiliations:** 1 Pulmonology Department, Hospital Universitari Germans Trias i Pujol, Carretera de Canyet S/N, 08916 Badalona, Barcelona, Spain; 2 Pathology Department, Hospital Universitari Germans Trias i Pujol, Badalona, Barcelona, Spain; 3 Medicine Department, Universitat Autònoma de Barcelona, Bellaterra, Barcelona, Spain; 4 CIBER de Enfermedades Respiratorias (CibeRes), Bunyola, Balearic Islands, Spain; 5 Pulmonology Department, Corporació Sanitària i Universitària Parc Taulí, Sabadell, Barcelona, Spain

**Keywords:** Cell block, Endobronchial ultrasound, Transbronchial needle aspiration, Lung cancer

## Abstract

**Background:**

Conventional smears of samples obtained by endobronchial ultrasound with real-time transbronchial needle aspiration (EBUS-TBNA) have proven useful in lung cancer staging, but the value of additional information from cell-block processing of EBUS-TBNA samples has only been marginally investigated. This study focussed on the contribution of cell block analysis to the diagnostic yield in lung cancer.

**Methods:**

Patients referred for lung cancer diagnosis and/or staging by means of EBUS-TBNA were enrolled, the adequacy of the obtained samples for preparing cell blocks was assessed, and the additional pathologic or genetic information provided from cell block analysis was examined.

**Results:**

In 270 lung cancer patients referred for EBUS-TBNA (mean age, 63.3 SD 10.4 years) 697 aspirations were performed. Cell blocks could be obtained from 334 aspirates (47.9%) and contained diagnostic material in 262 (37.6%) aspirates, providing information that was additional to conventional smears in 50 of the 189 samples with smears that were non-diagnostic, corresponding 21 of these blocks to malignant nodes, and allowing lung cancer subtyping of 4 samples. Overall, cell blocks improved the pathologic diagnosis attained with conventional smears in 54 of the 697 samples obtained with EBUS-TBNA (7.7%). Cell blocks obtained during EBUS-TBNA also made epithelial growth factor receptor mutation analysis possible in 39 of the 64 patients with TBNA samples showing metastatic adenocarcinoma (60.1%). Overall, cell blocks provided clinically significant information for 83 of the 270 patients participating in the study (30.7%).

**Conclusions:**

Cell-block preparation from EBUS-TBNA samples is a simple way to provide additional information in lung cancer diagnosis. Analysis of cell blocks increases the diagnostic yield of the procedure by nearly seven per cent and allows for genetic analysis in a sixty per cent of the patients with metastatic adenocarcinoma.

## Background

With the introduction of novel targeted therapies for non-small cell lung cancer (NSCLC), cytologists have had to cope with a corresponding rise in the need for accurate diagnosis and appropriate classification of subtypes. The analysis of genetic abnormalities in cancer cells, such as mutations in the epithelial grow factor receptor (EGFR) gene [1], has become crucial for the choice of treatment. Thus, conventional cytology staining does not always provide sufficient information and additional tissue is often required. The possibility of tailored treatments for lung cancer has come at the same time as the increased availability and use of minimally invasive sampling procedures, such as endobronchial ultrasound-guided transbronchial needle aspiration (EBUS-TBNA). This technique can obtain both mediastinal and hilar cytological samples of nodes and masses that are appropriate for conventional smear and, in most cases, for immunohistochemistry [2].

Material recovered during EBUS-TBNA can be processed additionally as a cell block and made available for ancillary diagnostic procedures. The usefulness of cell blocks has been acknowledged in fine-needle procedures, and several medical societies have recently recommended its routinely use for lung cancer diagnosis [3,4]. This processing technique, however, is not yet widely used on EBUS-TBNA and there is little information about its contribution to the diagnostic process. The aim of this study was to evaluate that contribution in a prospectively recruited series of patients undergoing EBUS-TBNA for the diagnosis and/or staging of lung cancer.

## Methods

### Population

In North Barcelona Health Area all patients who had a suspicion of lung cancer are referred by the general practitioner to the Lung Cancer Unit for diagnosis. EBUS-TBNA was used as a diagnostic procedure in patients with mediastinal masses and/or nodes and with negative results from previous endoscopic procedures. EBUS-TBNA was additionally used for staging in all NSCLC patients who did not show distant metastasis at the first examination. The present study included all lung cancer patients who were diagnosed and/or staged by means of EBUS-TBNA between January 2006 and December 2009. A CT scan of the lung, mediastinum, and upper abdomen was performed in all cases using a multidetector-row spiral CT scanner (Marconi M8000, Phillips, Best, The Netherlands) in the month prior to staging, and nodes with a short-axis diameter greater than 10 mm in the scan were considered abnormally enlarged [5]. EBUS-TBNA was used for staging in all referred patients, independently of the size of the nodes in the scan, in accordance with previous reports that have showed the usefulness of EBUS-TBNA for the diagnosis of mediastinal metastasis in patients with a normal-appearing mediastinum at CT [6]. Patients with hemorrhagic diseases or coagulation disorders were excluded from staging by TBNA. The research protocol was approved by the regional ethics committee (Institut de Recerca en Ciències de la Salut Germans Trias i Pujol, reference: FIS PS09/01612) and all patients gave their signed consent to participation

### EBUS-TBNA technique

EBUS was performed using a flexible bronchoscope (BF-UC160F-OL8, Olympus Optical Co Ltd., Tokyo, Japan) with a distal probe capable of producing linear parallel scans of the mediastinal and peribronchial tissues and a working channel suited to the performance of TBNA under direct ultrasound guidance. Local anaesthesia and conscious sedation were achieved using topical lidocaine spray and intravenous midazolam, respectively, in accordance with standard recommendations [7]. Mediastinal and lobar lung masses and nodes with a short-axis diameter of 5 mm or more [6] identified during the procedure were sampled under direct ultrasound visualization with a 22-gauge cytology needle specially designed for EBUS-TBNA (NA-201SX-4022, Olympus Optical Co Ltd.). The needle was guided beyond the bronchoscope channel to the tracheal lumen and then pushed forward from the sheath and inserted into the tracheal or bronchial wall under ultrasound guidance until the node or mass was reached. Once the needle tip was inside the target, negative pressure was maintained with a syringe at the proximal end of the catheter while the needle was pushed forth and back, releasing the suction before the needle was removed from the target structure.

### Pathology

The aspirated material in the needle was recovered and the specimens were placed on slides and fixed with 95% ethanol. The slides were stained 1 minute with haematoxylin for rapid on-site evaluation by a cytopathologist; later the Papanicolau staining with orange A and eosin was completed at the pathology laboratory. An immediate assessment was given after each pass. The cytologist classified nodes as "normal tissue negative for malignancy" when the sample contained 40 lymphocytes per high-power field in cellular areas of the smear and/or clusters of pigmented macrophages, and no neoplastic cells [8], or as "metastatic" when recognizable groups of malignant cells were present. Nodes containing only isolated dysplastic cells were considered as "suspicious" but non-diagnostic. Nodes containing only bronchial or blood cells, which were considered as not representative of the structure that was the target of the sampling procedure, were also classified a non-diagnostic. In these situations the procedure was repeated up to 3 times and considered as useful for staging only when diagnostic samples were recovered from at least one of the aspirates [9,10]. The obtention of neoplastic cells from one lower paratracheal or subcarinal (stations 4R, 4 L and 7) node during sampling diagnosed N2 or N3 disease and precluded the performance of additional samplings in these regions. Stations showing only nodes with a short-axis diameter less than 5 mm during EBUS-TBNA were not sampled and labelled as normal, in agreement with previously published results [6].

Cell blocks were obtained and processed from the specimens recovered in the first pass whenever extra clotting material was available after the preparation of a minimum of four slides, or from a second or third passes when clotting material for cell blocks was not obtained in the previous passes, at request of the on-site cytopathologist. Cell blocks were obtained air-drying and clotting the specimens on filter paper and then placing them into 10% formalin just after for subsequent processing in the laboratory [11]. Cell blocks were embedded in paraffin and sections of 5-μm thickness were obtained. Routine haematoxylin-eosin staining was used on cell-block sections and, when necessary, immunohistochemical stainings were applied for the identification or phenotyping of malignant cells. In cases of adenocarcinoma, somatic mutations of the genes coding the tyrosine kinase domain of EGFR were examined on cell-block samples, using methods previously described [12].

### Statistical analysis

Data were introduced in a database and analyzed using SPSS software version 17.0 (SPSS Inc., Chicago, Illinois, USA). Results were expressed as absolute and relative frequencies for categorical variables, and as means and standard deviations (SD) or, when required, as medians and interquartile ranges (IQR), for continuous variables. First, availability of cell blocks containing adequate tissue samples from nodes or masses sampled by means of EBUS-TBNA was assessed. Second, the provision of new pathologic information from these cell blocks was analyzed. Information additional to pathology was defined as the establishment of a cytological diagnosis through the examination of the cell block from a sample with a previous non-diagnostic conventional smear or the determination of the NSCLC subtype based on the cell block when the smear diagnosis was NSCLC not otherwise specified (NSCLC-NOS). Finally, the impact of the additional information provided by the analysis of cell blocks over patient staging was assessed. The recovery of a cell block suitable for performance of genetic analysis of EGFR mutations in patients with metastatic adenocarcinoma was considered as additional genetic information. A *p *value of 0.05 or less was reported as statistically significant in the performed statistical tests.

## Results

EBUS-TBNA was performed on 270 patients with a final diagnosis of lung cancer; the patient's mean age was 63.3 (SD 10.4) years and the male-to-female ratio was 6.7:1 (Table 1). EBUS-TBNA diagnosed metastasis in 130 out of 181 patients with evidence of enlargement in mediastinal nodes on the CT (71.8%), and in 14 of the 89 patients with a normal appearance of the mediastinum on the scan (15.7%).

**Table 1:** Population characteristics (n = 270)

Age, mean (SD), years	63.3 (10.4)
Gender (men), n (%)	235 (87)

Mediastinal nodal enlargement at CT, n (%)	181 (67.0)

Pathologic diagnoses, n (%)	

Adenocarcinoma	106 (39.3)

Squamous-cell carcinoma	65 (24.1)

Large cell carcinoma	10 (3.7)

NSCLC not otherwise specified	59 (21.8)

Small cell lung cancer	29 (10.7)

Atypical carcinoid	1 (0.4)

CT: computed tomography

NSCLC: non-small cell lung cancer

Of 697 TBNA procedures performed, with an average of 2.6 TBNA per patient, 672 aspirations were from nodes and 25 were from mediastinal masses. The median short-axis diameter of the sampled nodes was 10 mm (IQR 7-15) and 562 (80.6%) of them were in the mediastinum. Two-hundred twenty-three smears (32%) led to a diagnosis of metastatic disease, 285 (40.9%) showed lymphocytes and were negative for malignancy, 15 gave isolated atypical cells (2.1%) and 174 (25%) gave only non-representative material (Table 2).

**Table 2:** Diagnoses in conventional smears of transbronchial needle aspirates (n = 697)

Squamous cell carcinoma	29 (4.2)
Adenocarcinoma	98 (14.1)

NSCLC not otherwise specified	63 (9.0)

Small cell carcinoma	33 (4.7)

Normal tissue	285 (40.9)

Non-diagnostic	

Isolated atypical cells	15 (2.1)

Non-representative	174 (25.0)

NSCLC: non-small cell lung cancer

**Figure 1:**
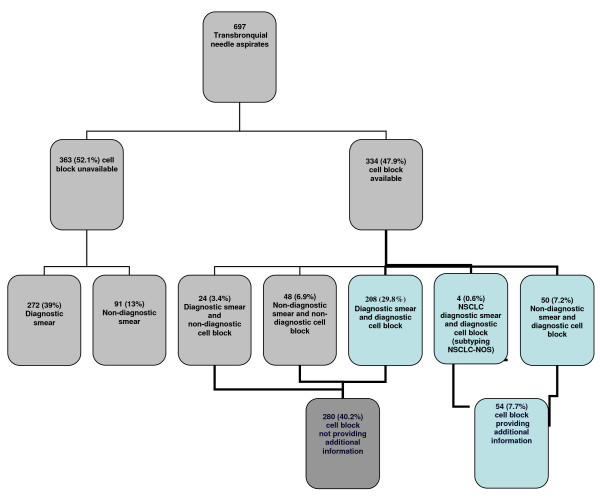
**Diagnoses from conventional smear and cell block obtained with endobronquial ultrasonography with transbronchial needle aspiration (NSCLC: non-small cell lung cancer)**.

Cell blocks could be prepared from 334 aspirates (47.9%) obtained from 321 nodes and 13 mediastinal masses and adequate material for diagnosis was recovered from 262 (37.6%) of them (Figure 1). The median short-axis diameter of nodes from which material for cell block processing was obtained was 11 mm (IQR 8-15), a size which was larger than the size of nodes that did not give material suitable for cell blocks after three passes (short-axis diameter 9 [IQR 7-14]) (*p *< 0.001, Mann-Whitney U test). Most of the samples with a cell block available were obtained from nodes located in the mediastinum, mainly in the subcarinal region (49.1%). Malignancy was diagnosed at the examination of 122 of the obtained cell blocks, being the block sample diagnostic and negative for lung cancer in 130 of the performed aspirations. In 10 cases the cell block showed only isolated atypical cells and was considered non-diagnostic.

Cell blocks provided additional pathologic information in 50 cases out of the 189 (26.4%) smears from samples that were non-diagnostic or that showed only isolated atypical cells (Figure 1). Twenty-one of these blocks corresponded to malignant nodes and 29 to normal nodes. Thus, information from cell blocks raised the overall diagnostic yield of EBUS-TBNA through an increase in the number of the diagnostic samples from 508 (72.9%) to 558 (80%). There were 63 cases of NSCLC-NOS on the conventional smear, in 4 (6.3%) of those the cell block achieved the subtype providing also additional pathologic information

Cell blocks obtained during EBUS-TBNA provided clinically significant information for 83 of the 270 patients participating in the study (30.7%). Pathologic diagnosis was attained in some nodes only through cell block processing in 40 patients (14.8%), and cell block was the only sample that demonstrated mediastinal metastases in 7 of them. In 4 patients with conventional smears showing NSCLC-NOS, cell blocks allowed the identification of the sub-type of the NSCLC. Additionally, cell blocks provided material suitable for EGFR gene mutation analysis in 39 of the 64 patients with metastatic adenocarcinoma in the sampled nodes (60.1%), and allowed the identification of a mutation of the EGFR gene in two patients.

## Discussion

Cell blocks prepared from EBUS-TBNA material in our series contained diagnostic material in a third of the samplings and provided additional information to non-diagnostic smears, increasing the accuracy of EBUS-TBNA by a seven percent, to a diagnostic yield of 80%. Cell blocks obtained during EBUS-TBNA provided clinically significant information for one third of the patients participating in the study (30.7%), through accurate typing of the disease, identification of metastasis in the mediastinum, and, in patients with adenocarcinoma, EGFR genetic analysis in cell block samples.

With the development of novel treatments for NSCLC that have different degrees of efficacy and toxicity in NSCLC subtypes, an accurate pathologic classification has become essential. Most patients with NSCLC present with advanced non-operable disease and surgical biopsies allowing additional pathologic and genetic analyses are not available [13]. The difficulties of pathologic diagnosis have increased with the emergence of minimally invasive procedures like EBUS-TBNA. This technique provides conventional smears for cytology that have a good correlation with histological diagnoses. Feller-Kopman and colleagues [14] compared the cytological samples obtained by EBUS-TBNA with core biopsies or surgical excision samples in a series of 88 patients, finding that diagnoses were equivalent in most patients. Cell blocks can be obtained by means of EBUS-TBNA, and, compared with conventional smears, allow the performance of sections suitable for larger immunohistochemical staining batteries [15,16]. When cell blocks prepared with EBUS-TBNA material are used for NSCLC subtyping, the adequacy of tumour tissue available for immunohistochemistry is a key issue [17]. That topic can be easily managed when the recovered samples are subject to rapid on-site evaluation, as in our study; thus the immediate evaluation of the sample increases the diagnostic yield and decreases the need for unnecessary repeated diagnostic procedures [18]. The on-site cytopathologist confirms the adequacy of the recovered material, minimizing the rate of unsatisfactory samples and requests for further sampling when additional material is needed for cell blocks. Following this approach 4 (6.3%) cases initially diagnosed as NSCLC-NOS on the conventional smear could be adequately subtyped in our study.

**Figure 2:**
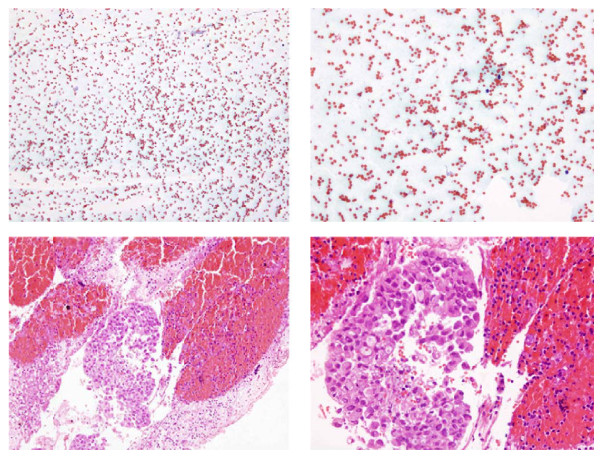
**Fine needle aspiration from a subcarinal node in a case of metastatic adenocarcinoma**. Non-diagnostic conventional smear (figure 2a and b) and cluster of adenocarcinoma cells in the the cell block (figure 2c and d). **a**: Unsatisfactory specimen. Blood cells in an otherwise acellular smear (Papanicolau stain, x 100). **b**: Unsatisfactory specimen. Blood cells in an otherwise acellular smear (Papanicolau stain, x 200). **c**: Cluster of adenocarcinoma cells in a cell block (Hematoxilin and eosin stain, x 100). **d**: Cluster of adenocarcinoma cells in a cell block (Hematoxilin and eosin stain, x 200).

We found that over a 75% of the recovered cell blocks contained diagnostic cellular material, a percentage similar to those in other series where cell blocks from needle core biopsies have been processed [3,19], but lower than the figure attained by conventional smears [18]. Cell-block analysis achieved the diagnosis in 50 cases out of the 189 samples (26.4%) in which conventional smears were non-diagnostic in our study. Thus, with cell-block processing, the diagnostic yield of EBUS-TBNA rose from 72.9% to 80%. Twenty-one of these diagnostic cell blocks were from malignant nodes that would not have been diagnosed if the blocks had not been obtained and clinically implies that 7 patients were diagnosed of mediastinal metastases (N2/N3 disease) solely by the cell block analysis. We attribute this increase in the diagnostic yield mainly to the contribution of cell blocks to haematic non-diagnostic smears (Figure 2). One of the obstacles that bronchoscopists and cytopathologists have to deal during an EBUS procedure is a vascularised node; these nodes are more likely to contaminate the samples with red blood cells. In this situation the on-site cytopathologist may not be able for a proper diagnosis of the slides. These aspirates, processed as cell blocks, can be examined later on the pathology laboratory and sometimes harbour clusters of lymphocytes or malignant cells. Other situation apart from blood contamination is nodes or masses containing necrotic material.

Cell-block processing allowed for the performance of EGFR mutational analysis in 60% of our patients with a diagnosis of metastatic adenocarcinoma and in two of them confirmed the presence of an EGFR mutation, which confer sensitivity to the tyrosine kinase inhibitors gefitinib and erlotinib [20]. These findings agree with the few smaller studies that have focussed on the ability of EBUS-TBNA to obtain samples for EGFR gene mutation screening [21,22]. Nakajima and cols. [21] used this approach in a series of 46 patients with adenocarcinoma, detecting 11 patients with EGFR mutations. García-Olivé and cols. [22] found nodal metastasis by means of EBUS-TBNA in 36 patients from a series of 51 patients with this diagnosis; these authors recovered cell blocks that were adequate for EGFR analysis through EBUS-TBNA for most of their patients, and were able to identify mutations in two of them. Other cancer-related genetic mutations may also be predictive biomarkers, and their detection in TBNA samples might be useful for choosing a lung cancer therapy [1]. In this new scenario our study confirms the value of cell-block processing of the material recovered from malignant nodes using EBUS-TBNA.

In summary, cell-block preparation is a simple method that provides important additional information after EBUS-TBNA in lung cancer. In our study, it was possible to preserve diagnostic material for cell blocks from more than a third of the performed aspirates. This material supplemented the information from conventional smears in a third of the cases and increased the diagnostic yield of the technique by a seven percent. Overall, cell-block processing provided clinically significant information for on third of the lung cancer patients, and allowed for the performance of genetic analyses of EGFR mutations in a half of the samples showing metastatic adenocarcinoma, confirming the advantages of this processing method for the diagnosis and staging of lung cancer.

## Competing interests

The authors declare that they have no competing interests.

## Authors' contributions

JSS performed EBUS-TBNA, analyzed the data and wrote the original. PS performed EBUS-TBNA and acquired the data FA performed EBUS-TBNA and revised the final text. EC carried out the cytological examination. MLL carried out the cytological examination. EM performed EBUS-TBNA, designed the study and revised the final text.

## Pre-publication history

The pre-publication history for this paper can be accessed here:

http://www.biomedcentral.com/1471-2407/12/34/prepub
